# Monoclonal Gammopathy of Renal Significance with Progression to Multiple Myeloma in a Patient with ASIA-MO Syndrome

**DOI:** 10.1155/2022/8571536

**Published:** 2022-05-24

**Authors:** David Alejandro Vargas Gutiérrez, Karina Ivonne Arias Callejas, Edwin Pavel Palacios Ruiz, Priscila Joseline Pérez Vinueza, Juan Diego Muñoz Vega, Ana Karen G. Mejía Geraldo, Ingrid Salinas Zaldívar

**Affiliations:** ^1^Department of Rheumatology, Instituto Nacional de Rehabilitación Luis Guillermo Ibarra Ibarra, CDMX, Mexico; ^2^Department of Rheumatology, Instituto Nacional de Cardiología Ignacio Chávez, CDMX, Mexico; ^3^Department of Internal Medicine, Hospital General de México: “Dr. Eduardo Liceaga”, CDMX, Mexico; ^4^Department of Hematology, Hospital General Mexico: “Dr. Eduardo Liceaga”, CDMX, Mexico; ^5^Department of Pathological Anatomy, Hospital General Mexico: “Dr. Eduardo Liceaga”, CDMX, Mexico

## Abstract

**Background:**

Autoimmune/inflammatory syndrome induced by adjuvants is a disease associated with an unregulated hyperactivity of the immune system and may also be associated with a high frequency of hematologic malignancies. *Report*. This is a case of a female with ASIA-MO syndrome secondary to infiltration of mineral oil for aesthetic purposes and presented with multiple episodes of urolithiasis resulting in renal impairment of her left kidney confirmed by scintigraphy and ending in unilateral nephrectomy. Retrospective renal piece analysis confirmed tubulointerstitial infiltration with light chains and plasma cells. Paraffin fixation prevented subsequent immunofluorescence analysis for better follow-up of the patient.

**Conclusion:**

The presence of positive immunofixation kappa chains explained the sudden deterioration of renal function with monoclonal gammopathy of renal significance which concluded in an association between diseases, such as multiple light chain myeloma, as a final diagnosis.

## 1. Introduction 

This case report presents a patient with end-stage renal disease and hypercalcemia of unknown etiology alongside with a long-standing inflammatory state.

Therefore, hypercalcemia malignancy suspicion was intensified by identifying parathormone (PTH) suppression, in addition to light-chain screening with a positive monoclonal gammopathy and a bone biopsy with 60% of clonal plasma cells receiving a diagnosis of multiple myeloma with an ominous outcome.

Because of the absence of risk factors for a young female with end-stage renal disease, multiple etiologies were subjected to analysis such as monoclonal gammopathy of renal significance (MGRS), amyloidosis, and nephrocalcinosis, among others.

As a consequence, it was decided to review the surgical piece postmortem with light-chain deposit, tubular damage, and light-chain proximal tubulopathy (LCPT) as the main findings.

## 2. Case Presentation

This is a case of a 38-year-old female from the Mexico City with a history of infiltration of mineral oil in the mammary region back in 2001, which conditioned the development of subcutaneous nodules, thickening of the skin, and hyperpigmentation. This lesion spread throughout the anterior chest and abdomen, resulting in mechanical lumbar discomfort.

Her condition began one year before her first hospital admission with multiple episodes of urinary tract infection (UTI) secondary to urolithiasis which required left nephrostomy with subsequent clinical improvement. She was diagnosed with renal lithiasis, along with hypercalciuria (urinary calcium 322 mg/dl), CaU/CrU = 0.33, and phosphorus excretion fraction of 21%, and it was initially managed with thiazide diuretic (hydrochlorothiazide).

However, 6 months later, fever returned together with a fall in the glomerular filtration rate (GFR) of 44 ml/min/1.73 m^2^ CKD-EPI (Chronic Kidney Disease Epidemiology Collaboration) (Table 1), but also proteinuria in the subnephrotic range (960 mg/24 hours), albuminuria of 53 mg/24 hours, and urinary sediment with granular casts, without dysmorphia or acanthocytes. Blood tests reported hypoalbuminemia (3.2 mg/dl) and total serum protein of 7.8 mg/dl. Renal USG showed bilateral loss of the medullary cortex relationship and a left kidney size of 9.6 cm^*∗*^4.4 cm^*∗*^45.2 cm (without pyelocaliceal dilation or any anatomy abnormalities); however, it did describe stranding of the perinephric fat on the right kidney as a sign of pyelonephritis.

As part of the febrile syndrome workout, immunological tests were performed such as antinuclear and antineutrophil cytoplasm antibodies; both were negative. After a myeloproliferative syndrome was suspected, the BMA smear was labeled as normocellular without the presence of blasts, with plasma cells <7%, and with negative myelocultures. Treatment with empirical antimicrobials showed improvement in the infection and fever of unknown origin.

Unfortunately, 3 months later, the renal function continued to deteriorate, requiring renal scintigraphy, reaching a GFR of 10.1 ml/min in the left kidney and 61 ml/min in the right one, determining renal exclusion. Due to recurrent pyelonephritis and the exclusion mentioned above, a left nephrectomy was performed. Despite this approach, 3 months later, the patient presented with pallor, palpitations, fatigue, nausea, and persistent vomiting, and blood work reported acidosis and a high anion gap (16), accompanied by azotemia, normocytic normochromic anemia (hemoglobin 7 mg/dl), and moderate hypercalcemia (corrected calcium 12.7 mg/dl).

**Figure 1:**
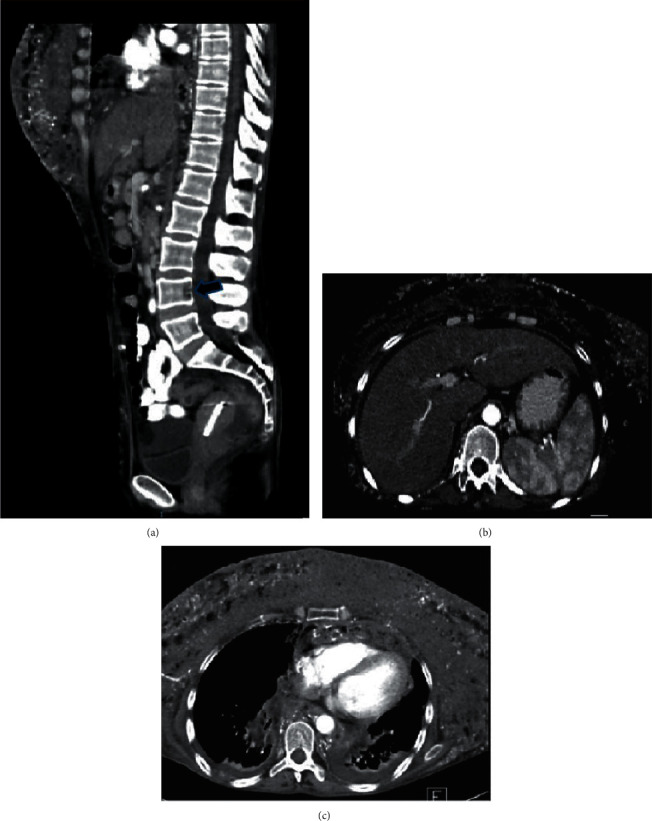
Computed axial tomography (contrast phase). (a) Sagittal section shows an increase in soft tissue volume in the mammary region of the L4 vertebral body. A hypodense lesion with continuity loss is seen at the posterior vertebra wall suggestive of erosive injury (arrow). (b) Cross section at the abdomen shows the spleen with granular characteristics and heterogeneity of its content. (c) In the thorax, abundant granular tissue is observed not only at the rib cage but also in the anterior and posterior mediastinum, secondary to the substance dissemination and infiltration.

She required hemodialysis, IV fluids, diuretic therapy, and blood transfusion with clinical improvement. Despite this, hypercalcemia persisted with PTH suppression (1.8 pg/ml). Consequently, the analysis was redirected for independent PTH hypercalcemia, ruling out 25-hydroxycholecalciferol toxicity (24 ng/dl) (she actually had this deficiency). Phase-contrast CT showed a large-volume granulomatous disease with extensive infiltration which included the anterior and posterior mediastinum, pelvis, and spleen. Moreover, evidence of splenomegaly in the absence of tumor disease and lytic lesions is found in the L4 posterior wall (Figure 1). Abnormally high concentrations of *β*2 microglobulin (B2M) (5.6 mg/L) were found, which could also be found not only in lymphoproliferative disorders but in renal failure as well (41). Serum immunoglobulin was normal, but high values of serum-free kappa light chains (FLCs) (102 mg/L) and the lambda chain (9.1 mg/L) were additionally with a *κ*/*λ* ratio (11.2) as synonymous to monoclonality. Urinary and serum protein electrophoresis was analyzed without evidence of any gamma monoclonality.

Urinary protein immunofixation electrophoresis (IFE) showed tubular protein positivity with a monoclonal kappa band peak along with anemia, hypercalcemia, and renal function impairment of unknown etiology pointing to plasma cell dyscrasia. Bone biopsy showed clonal plasma cells (immunohistochemistry with 60% positive plasma cells for CD38).

Then, MGRS evolving into MM was concluded, and chemotherapy sessions were scheduled. However, by yielding a poor result, association of ASIA-MO and kidney disease reflected in the death of the patient, even before we could start the procedure.

**Figure 2:**
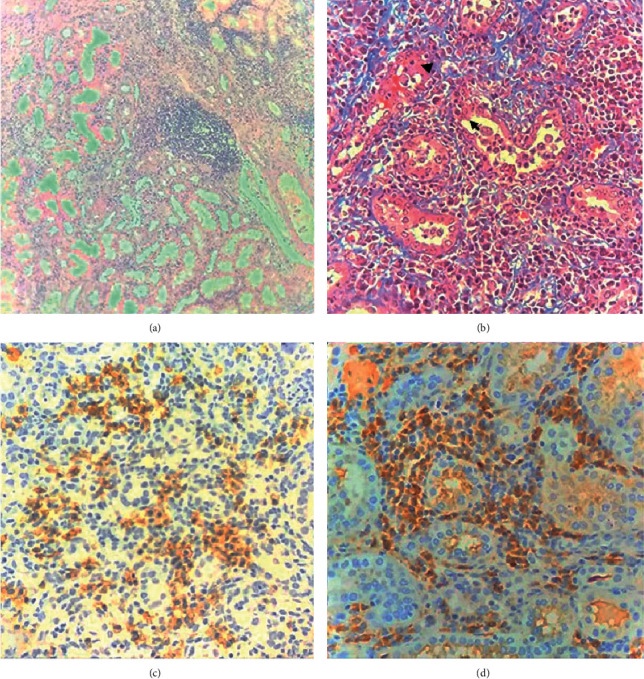
Renal biopsy immunohistochemistry. (a) In the microphotograph with HyE, we can observe part of the medulla composed by tubules with acute tubular necrosis and disappearance of nuclei in a few cells. The interstitium is altered by the presence of the chronic lymphoplasmacytic infiltrate, arranged by cumulus in some areas, but also the presence of slight fibrosis. (b) Masson staining shows interstitial thickening secondary to the lymphoplasmacytic infiltrate reaching the proximal contoured tubules giving an irregularity in its contour. As a paramount feature, the presence of eosinophilic inclusions (arrowhead) in the cytoplasm of the epithelium covering these tubules is seen and also the desquamation of this epithelium which is compatible with LCPT (arrow). Blue staining confirms the presence of slight fibrosis. (c) Immunohistochemical staining for CD138 (plasma cell biomarker) was performed with a significant reaction at the membrane level highlighting the abundant presence of plasma cells. (d) Reaction to kappa light chains (brown staining) shows variable positivity inside the cytoplasm of plasma cells at the interstitium, which represents its monoclonality.

In conclusion, a postmortem histopathological of the renal piece reported the presence of interstitial nephritis with the plasma cell infiltrate with positive immunohistochemistry for light-chain kappa in the interstitium and LCPT (Figure 2).

## 3. Discussion

### 3.1. Monoclonal Gammopathy of Renal Significance and LCPT

Paraprotein originates from monoclonal immunoglobulins or their light chains, characterized by the expression of a single light chain, *κ* or *λ*, also called monoclonal protein or M protein. The most common is the low-level nonmalignant paraprotein monoclonal gammopathy of undetermined significance (MGUS) [1].

Approximately 1% of asymptomatic MGUS (IgG or IgA) will progress to MM, which could later become light-chain MM or AL amyloidosis. Currently, this “indolent” state is considered premalignant, although it has been questioned since cloned plasma cells may occasionally be responsible for serious organ damage through tissue toxicity by M protein [2].

MGRS is defined as a plasma or lymphoplasmocytic clonal cell disorder with secretion and deposit of monoclonal immunoglobulins or their fractions in the renal parenchyma with toxic effects by several mechanisms [3].

This kidney injury phenomenon was replicated in animal models where Bence Jones proteins were administered intravenously or by intraperitoneal infusion, resulting in deposit-associated nephropathy which supports the theory that MM presence is not always required [4].

In chronic glomerular injury, tubular cells lose integrity with an increase in proinflammatory cytokines. Moreover, patients with higher amounts of urinary protein >20 KDa presented poor response rates to both glucocorticoid and cyclophosphamide treatments than patients with proteinuria <20 KDa alone (30% vs. 80%) such as light chains [5].

MGUS development is multifactorial. The most accepted theory states 2 essential hits in the development of premalignant and malignant lesions; the first one is an abnormal response to antigenic stimulation, mediated by an aberrant expression of TLRs, and the second one is determined by overexpression of IL-6r and IL-1r secondary to persistent inflammatory processes as observed in our patient, which promoted the development of primary cytogenetic abnormalities. The development of MM manifestations from premalignant states such as MGUS or SMM requires a second DNA lesion or loss of cell cycle surveillance and repairing systems such as chromosome 16 methylation, MYC abnormalities, and RAS and p53 mutations [6].

Therefore, symptoms of MM could be initially noticed after nephrectomy, expressed by low levels of plasma cells with dysplasia [7]. A high incidence of malignant tumors is observed in CKD due to the chronic increase in cytokines and growth factors caused by hyperazotemia, low renal clearance of proinflammatory cytokines, and oxidative stress [8]. Furthermore, high levels of sialic acid are present in the monoclonal immunoglobulins of MGUS and MM [9], and this was observed in the analysis of 2817 CKD patients in a hospital in Turkey, with the report of malignancy on 6.7% of patients (188) and 2.6% of MM [10].

In multiple myeloma, the serum level of *β*2M has been widely studied as a biomarker of renal function in humans and murine models; however, it can increase its production and its presence as a free molecule in hematological and solid neoplasms. Part of this is the main determinant in the International Staging System (ISS), which predicts not only the prognosis but also the progression of asymptomatic disease (HR 3.30; *P*=0.002); despite this, it is important to take into account that renal insufficiency can elevate *β*2M levels and therefore complicates determination of the exact origin of an elevated serum level [11].

While kidney damage in the presence of intact immunoglobulin molecules is limited to the glomerulus, incomplete light and heavy chains can affect all renal compartments, but tubulointerstitial lesions are caused by any of the light chains (most often kappa). Light chains are internalized by tubular epithelial cells; when this tubular reabsorption capacity is exceeded, the intracellular protein gradient saturates, resisting degradation with the generation of ROS, sustained inflammation, and apoptosis of epithelial cells.

LCPT is an uncommon form of renal disease associated with progressive renal failure dysproteinemia with the presence or absence of inclusion. It presents with a normal glomerulus along the first needle-shaped rhomboidal or rectilinear eosinophilic inclusions (periodic acid-Schiff (PAS) negative) by light microscopy, and LCPT without inclusion and vacuolar changes or excessive drops, necrosis of epithelial cells with displacement, detachment, and apical blisters are common findings with 13%–77% [12, 13].

### 3.2. ASIA-MO

ASIA is a disorder associated with unregulated hyperactivity of the immune system with the infiltration of exogenous substances as a main characteristic. The initial criteria proposed in 2011 by Shoenfeld and Agmon-Levin to classify patients with autoimmune manifestations with preexisting conditions together with adjuvants have generated controversy. Despite that, the so-called ASIA associated with mineral oil (ASIA-MO) was added recently to this classification and defined as the infiltration of modeling substances for aesthetic purposes. Mineral oils (MOs) are saturated hydrocarbons of variable, linear, and branched ring chain structures of carbon. The MO acts as an adjuvant capable of accelerating, prolonging, or increasing an autoimmune response [14].

High incidence of systemic manifestations of autoimmune-type phenomena, such as carbon-rich mineral oils and silicone, has shown to promote animal model expansion of the adaptive immune response, producing ASIA-MO-like syndromes [15].

Isolated cases of ASIA-associated breast cancer reported with the highest frequency of hematologic malignancies such as non-Hodgkin lymphoma [16, 17]. Anaplastic large-cell lymphoma associated with breast implants is a recent clinical entity with 80 cases identified worldwide and four associated deaths. In 2016, the WHO classified this subtype as a recognized entity and emphasized the importance of foreign material removal surgery as a prophylactic measure [18]. The cytogenetic and biochemical study of 3 patients with breast anaplastic lymphoma and breast implants was described by Lechner et al., showing an increase in STAT3 signaling related to the autocrine production of IL-6, a mechanism shared with the development of MM [19]. In contrast, a study by Karlson et al. found little evidence for MGUS risk in patients with breast implants [20].

Anderson and Potter demonstrated the molecular association of plasma cell neoplasms and ASIA-MO in BALB/c susceptible murine models with pristane intraperitoneal induction as an enhancer of the innate immune system with arthritis, lupus-related autoantibodies, and IgA-secreting plasma cell neoplasms as a result [21, 22]. However, it also relates to the granulomatous reaction at the site of application with limited tissue antigen clearance, granulomatous body strange reaction, and a proinflammatory state [23]. Other studies observed small plasmocytomas within 3 weeks of subsequent infiltration with kappa or lambda polypeptides which induce nephrosis [24].

### 3.3. Diagnostic Approach

Low levels of vitamin 25 (OH) D, related to the CKD, and hypercalcemia of malignancy contributed to decreased reabsorption and higher excretion of calcium. In this particular case, the presence of granulomatous disease in thoracic visceral sites was demonstrated by CT with ASIA-MO-induced spread and initial inflammation in both breasts.

Granulomatous pathologies in the breast are complex problems that require an exhaustive study to identify various causes, including infectious etiologies such as mycobacterial infection (*M. tuberculosis*). In this patient, IGRAs screening was negative. Other autoimmune etiologies of granulomatous mastitis are sarcoidosis, which represents 1% of these disorders, and GPA. The first was ruled out because it did not present classic sarcoid conditions in other sites and vitamin D levels were low. GPA was ruled out since it did not show evidence of epithelial proliferative lesions of the glomerulus, and ANCA antibody titers were reported negative. Finally, hematological neoplasms such as lymphoma were ruled out given that during its evolution, it did not present B symptoms, although no biopsy of granulomatous or ganglion tissue was performed [25]. Finally, systemic amyloidosis AA or AL was ruled out in the absence of nephrotic phenotype, and the biopsy was Congo red negative.

Another mechanism carrying tubular damage is the presence of hypercalcemia with hypercalciuria but also with precipitation of calcium crystals and lesions of the epithelium affected by episodes of lithiasis. Bataille et al. evaluated osteoclast-mediated bone resorption in 87 bone biopsies from patients with MGUS, 45% of whom presented some degree of bone resorption and 52% of whom were classified as high-risk patients which progressed to MM compared to 4% of the low-risk group [26]. Low-grade bone resorption through premalignant or malignant neoplasms of plasma cells reflected at the urinary level by hypercalciuria since compensation mechanisms manage to mitigate the increase in calcium plasma levels as observed in our case report [27].

Autoimmune diseases along with chronic inflammatory conditions are reported to be associated with an increased risk of MGUS [28]. Malignant transformation is associated with the presence of heavy-chain IgA, high levels of ESR, high percentage of plasma cells in the bone marrow, and osteoporosis [29]. A case-control study involving men and women diagnosed with MM and MGUS over a period of 40 years found an increased risk of MGUS (OR = 2.1; 95% CI, and 1.9–2.4) in patients with all subcategories of autoimmune diseases characterized by chronic inflammation (OR = 1.4; 95% CI, 1.2–1.5) [30].

Yang et al. describe 41 patients with rheumatic diseases and a positive screening for monoclonal gammopathies, the most common associated finding being Sjоgren's syndrome (SSj). Disease activity was an independent risk factor for the development of monoclonal gammopathy, and its presence was associated with an OR of 17.5 (95% CI: 1.5–197) for MM. Similar findings in other autoimmune diseases such as rheumatoid arthritis, systemic lupus erythematosus, and inflammatory myopathies with MM or MGUS had an RR of 2.29 with a prevalence of MGUS up to 42%. Here, chronic B cell activation and antibody production develop a chronic immune environment with pro-oncogene mutations [31].

Chronic inflammatory nephropathy previously reported by pathologists was a light-chain nephropathy; hence, immunohistochemistry was performed with kappa chain findings and changes suggestive of LCPT. Despite the current awareness of ASIA-MO syndrome, there is still skepticism that this could explain why this patient was not clearly diagnosed or treated for 18 years; as a consequence, disease activity could become a possible explanation.

## 4. Conclusion

This is an interesting case because it describes the possible association between both pathologies in which chronic inflammation becomes a substrate triggered by ASIA as an epigenetic factor aimed at monoclonal paraproteinemia, tubular injury, and acute inflammation, where the nephrectomy played a second hit which contributed to the final manifestation of MM. There are not enough studies in the field, and there is much heterogeneity which makes it difficult to analyze this or any other case. We suggest that more studies should be analyzed in the future, and hopefully, prospective studies will be conducted to determine the relationship between these two pathologies.

## Figures and Tables

**Table 1:** Laboratory and clinical findings in a chronological order.

Parameters	Initial 03/06/2016	Nephrectomy 01/08/2017	Reentry for impairment 01/04/2018
Urea (mg/dl)	36	51	290
Creatinine (mg/dl)	1.5	2	7.1
Uric acid (mg/dl)	7.1	8.5	6
Total proteins (mg/dl)	5.2	7.7	6.9
Albumin (mg/dl)	1.5	2.17	2.3
Corrected calcium (mg/dl)	10.9	12.7	13.2
Phosphorus (mg/dl)	3.6	3.2	4
WBC (cel/L)	15,000	6.5	7.6
Neutrophils (cel/L)	13,300	4.8	5.3
Hemoglobin (g/dl)	10.4	9	7
MCV (fL)	90	83	96
MCH (pg)	32	30	33
Platelets (109/L)	410	373	348

WBC: white blood cell, MCV: mean corpuscular volume, and MCH: mean corpuscular hemoglobin.

## Data Availability

The resources for this study come directly from the patient's medical record.

## Abbreviations

ASIA: Autoimmune/inflammatory syndrome induced by adjuvants
ASIA-MO: ASIA associated with mineral oil
BMA: Bone marrow aspirate
B2M: *β*2 Microglobulin
CKD-EPI: Chronic Kidney Disease Epidemiology Collaboration
CT: Computed tomography
DAMPs: Molecular patterns associated with damage
DC: Dendritic cells
ESR: Erythrocyte sedimentation rate
FLCs: Free light chains
GFR: Glomerular filtration rate
IFE: Immunofixation electrophoresis
IL-6r: Interleukin 6 receptor
LCPT: Light-chain proximal tubulopathy
MGRS: Monoclonal gammopathy of renal significance
MGUS: Monoclonal gammopathy of undetermined significance
MM: Multiple myeloma
PAS: Periodic acid-Schiff
PTH: Parathormone
ROS: Reactive oxygen species
SSj: Sjоgren's syndrome
SMM: Smoldering multiple myeloma
TLRs: TOLL-like receptors
UTI: Urinary tract infection.
